# Early Driving Pressure Is Associated with Major Adverse Kidney Events at 30 Days in ARDS Patients with SARS-CoV-2

**DOI:** 10.3390/jcm14082783

**Published:** 2025-04-17

**Authors:** Gustavo Casas-Aparicio, Adrián E. Caballero-Islas, Antonio León-Ortiz, David Escamilla-Illescas, Yovanna Rueda-Escobedo, Carlos Ascención-López, Diana Hernández-Quino, Aimee Flores-Vargas, Jesús Sosa-Chombo, Abraham Tolentino-de La Mora, Ana Saucedo-Pruneda, Elvira Piten-Isidro

**Affiliations:** 1 Coordinación de Nefrología, Instituto Nacional de Enfermedades Respiratorias “Ismael Cosío Villegas”, Calzada de Tlalpan 4502, Ciudad de México 14080, Mexico; adrian.caballero24@gmail.com (A.E.C.-I.); md.jaleon@gmail.com (A.L.-O.); 2 Departamento de Investigación en Enfermedades Infecciosas, Instituto Nacional de Enfermedades Respiratorias “Ismael Cosío Villegas”, Calzada de Tlalpan 4502, Ciudad de México 14080, Mexico; elvira.piten@cieni.org.mx; 3 Dirección de Medicina, Fundación Clínica Médica Sur. Puente de Piedra 29, Col. Toriello Guerra, Ciudad de México 14040, Mexico; davidei.md@gmail.com; 4 Departamento de Enseñanza, Instituto Nacional de Enfermedades Respiratorias Ismael Cosío Villegas, Calzada de Tlalpan 4502, Col. Sección XVI, Ciudad de Mexico 14080, Mexico; ymre90@gmail.com; 5 Facultad de Medicina Benemérita Universidad Autónoma de Puebla, Heroica Puebla de Zaragoza 72420, Mexico; carlos.ascencion.cieni@gmail.com (C.A.-L.); diana.hernandez@cieni.org.mx (D.H.-Q.); 6 Médico Adscrito a la Subdirección de Atención Médica de Neumología, Instituto Nacional de Enfermedades Respiratorias Ismael Cosío Villegas, Calzada de Tlalpan 4502, Col. Sección XVI, Ciudad de Mexico 14080, Mexico; aimefloresvargas@gmail.com (A.F.-V.); sosachombo@gmail.com (J.S.-C.); ana.s.pruneda@gmail.com (A.S.-P.); 7 Departamento de Investigación en Tabaquismo y EPOC, Instituto Nacional de Enfermedades Respiratorias Ismael Cosío Villegas, Calzada de Tlalpan 4502, Col. Sección XVI, Ciudad de México 14080, Mexico; dr.a.tolentino@outlook.com

**Keywords:** COVID-19, driving pressure, N-Gal

## Abstract

**Background:** Major adverse kidney events (MAKEs), including death, persistent AKI (pAKI), and renal replacement therapy, are more common in SARS-CoV-2-related ARDS. Invasive mechanical ventilation (IMV), systemic inflammation, and hemodynamic changes drive this risk. This study examines early IMV settings and urinary kidney biomarkers (UKBs) to better understand the development of MAKEs at 30 days. **Methods:** This prospective, cross-sectional cohort study was conducted in a single center between September and October 2021. This study included adults (≥18 years) diagnosed with ARDS due to SARS-CoV-2, requiring IMV within the first 6 h of admission. Exclusion criteria included a history of chronic kidney disease (CKD) and pregnant women. Initial mechanical ventilator settings were recorded after compliance-guided PEEP titration, and urine samples were collected for the analysis of UKBs at the same time. Our primary and secondary endpoints were to assess risk factors associated with MAKEs at 30 days and pAKI, respectively. **Results:** The cohort included 45 patients, with a median age of 57.75 (±18.64) years. In total, 32 (71%) developed MAKEs and 22 (48.8%) developed pAKI. MAKEs were associated with older age (adjusted odds ratio (aORs) = 1.23 95% CI: 1.00–1.22; *p* = 0.038) and higher driving pressure (ΔP) (aORs = 1.62, 95% CI:1.01–2.60, *p* = 0.043). Only urinary neutrophil gelatinase-associated lipocalin (uNGal) > 40 ng/mL was associated with pAKI (aORs = 8.54, 95% CI:1.75–41.65, *p* = 0.008). **Conclusions:** Early ventilator settings, particularly higher ΔP, play a critical role in the development of MAKEs. uN-Gal could enhance the early detection of pAKI, providing opportunities for timely interventions.

## 1. Introduction

Adults with severe acute respiratory distress syndrome (ARDS) due to SARS-CoV-2 have faced high mortality rates, with acute kidney injury (AKI) emerging as a significant contributor to these outcomes [1,2,3]. Major adverse kidney events (MAKEs), a composite outcome encompassing death, progression to persistent AKI (pAKI), and the need for renal replacement therapy (RRT), are more prevalent in patients with ARDS secondary to SARS-CoV-2 compared to other forms of ARDS [2,3].

The risk of MAKEs in ARDS patients is driven by the combined effects of mechanical ventilation, systemic inflammation, and hemodynamic changes [4]. The initiation of invasive mechanical ventilation (IMV) can accelerate the progression of these outcomes due to increased hemodynamic instability from ventilator pressures and the inflammatory response associated with ventilator-induced lung injury (VILI) [5,6,7,8,9]. The early phase of IMV is therefore crucial, as it carries the potential for adverse interactions between the respiratory and renal systems [5,10,11].

To enhance the early detection of patients at risk of adverse renal events, various initiatives advocate for establishing specific goals to improve early recognition and timely response, thereby mitigating further damage [12]. In this way, several biomarkers related to kidney damage [13], cell cycle arrest [14], and renal tubular repair [15] proved to be effective in predicting the severity and progression of AKI in patients with severe COVID-19 pneumonia.

In this context, we designed a study focusing on the initiation of IMV, a critical moment for patients with ARDS secondary to SARS-CoV-2. Following ventilator adjustments using PEEP-guided titration, we recorded the mechanical ventilation parameters and simultaneously collected a urine sample to measure the biomarkers N-Gal and TIMP-2/IGFBP7, biomarkers indicative of kidney structural damage, to deepen our understanding of renal injury and improve our understanding of the development of MAKEs in patients with ARDS and SARS-COV-2.

## 2. Material and Methods

### 2.1. Study Population

This prospective study was conducted at the National Institute of Respiratory Diseases (INER) with the approval of the INER ethics committee under the number C26–20. From this approval, two cohorts were established: one longitudinal cohort, which has already been published [14], and the current cross-sectional cohort with a different set of patients.

Participants over 18 years old who required mechanical ventilation within the first 6 h of admission to the emergency room and provided informed consent were included. Pregnant women and those with a history of chronic kidney disease were excluded. ARDS was diagnosed using the Berlin Criteria, and SARS-CoV-2 infection was confirmed by a positive PCR test from a nasopharyngeal swab [16,17].

The primary outcome was to determine the factors associated with the development of MAKEs at 30 days. The secondary outcome was to identify the factors associated with the development of pAKI during the first 48 h after the initiation of mechanical ventilation. Recorded variables included demographic and anthropometric variables, symptoms, comorbidities, critical care variables, blood chemistry, blood count, starting and termination dates of IMV, days in hospital, use of vasoactive drugs, and outcomes. Initial mechanical ventilator settings were recorded after compliance-guided positive end-expiratory pressure (PEEP) titration, which is a strategy based on the best respiratory system compliance. This approach aims to identify the PEEP level at which alveolar recruitment is optimized, avoiding both overdistension and collapse. Titration was conducted either in ascending or descending fashion (from 5 to 20 cmH_2_O in 2 cmH_2_O steps), maintaining each level for 1 to 2 min. At each step, dynamic compliance (tidal volume/[peak airway pressure − PEEP]) and static compliance (tidal volume/[plateau pressure − PEEP]) were measured. The PEEP level associated with the highest compliance was considered the optimal point of mechanical efficiency. A decrease in compliance with increasing PEEP was interpreted as a sign of overdistension [18].

Ventilatory parameters were analyzed at three distinct time points. The initial measurement (time 0) was obtained immediately after the initiation of invasive mechanical ventilation and corresponded to the PEEP titration procedure described above. Two additional time points—at 48 h and 120 h—were derived from the daily ventilator parameter records collected during routine morning rounds (between 6:00 and 7:00 a.m.).

### 2.2. Definition of MAKEs and Persistent AKI

We defined MAKEs as a composite outcome that includes in-hospital death, persistent acute kidney injury (pAKI), or the need for acute renal replacement therapy (RRT) [2]. pAKI was defined as continuance of AKI by serum creatinine beyond 48 h according to the consensus report of the ADQI 16 Workgroup [19].

### 2.3. Determinations of Urinary Renal Stress Biomarkers

Biomarker measurements were performed on urinary samples collected after compliance-guided PEEP titration. The samples were frozen at −80 °C within 30 min of collection. Urinary levels of the tissue inhibitor of metalloproteinases-2 (TIMP-2) and insulin-like growth factor binding protein 7 (IGFBP7) were measured using commercially available ELISA kits (Human TIMP-2 Quantikine ELISA Kit, R&D Systems, Minneapolis, MN; Human IGFBP7 ELISA Kit, Abcam, Cambridge, UK), according to the manufacturer’s instructions. ELISA plates were read at an optical density of 450 nm, and calculations were based on the standard curves provided by each kit. Neutrophil gelatinase-associated lipocalin (NGAL) concentrations were determined using the NGAL kit (Abbott, Chicago, IL) following the manufacturer’s protocol on the Abbott™ ARCHITECT™ Analyzer. Additionally, urinary interleukin-6 (IL-6) levels were measured using the Human IL-6 High Sensitivity ELISA Kit (Catalog: BMS213-2HS, Invitrogen), in accordance with the provided instructions.

### 2.4. Statistical Analysis

Continuous variables were expressed as mean and standard deviation (SD) for normally distributed data, and as median with interquartile range (IQR) for skewed data. Categorical variables were presented as counts (percentages). Descriptive statistics were compared using the χ^2^ test for categorical variables and the Mann–Whitney U test for continuous variables. To evaluate the diagnostic value of urinary N-Gal levels, receiver operating characteristic (ROC) curves were generated, and the area under the curve (AUC) with 95% confidence intervals was calculated at various cut-off points. The optimal cut-off point was determined by balancing sensitivity and specificity using samples collected after PEEP titration. Logistic regression was employed to identify associations between relevant covariates and major adverse kidney events (MAKEs) at 30 days, as well as persistent acute kidney injury (AKI). No violations of assumptions were detected. Multivariate models were constructed using a stepwise procedure, including variables with a *p*-value < 0.20 in univariate analysis. Age and gender were included in the models regardless of the alpha level. All statistical tests were two-tailed, with a *p*-value < 0.05 considered statistically significant. To assess the robustness of our findings given the limited sample size, we conducted a sensitivity analysis. This included an outlier assessment through the visual inspection of variable distributions and a multivariable bootstrapping analysis (5000 resamples) adjusting for driving pressure (ΔP), age, and sex.

Cumulative MAKE–30 days curves compared patients between groups of ΔP > 14 cmH_2_O vs. ΔP < 14 cmH_2_O using the Kaplan–Meier method and compared by the log-rank test. We use a ΔP > 14 cmH_2_O, as a cut-off, aligning with the findings from the LUNG-SAFE study, which did indeed associate higher ΔP with increased mortality in ARDS patients [20]. All the analyses were conducted using SPSS v 27.0.

## 3. Results

### 3.1. Characteristics of Study Participants

Between September 2021 and October 2021, a total of 123 individuals were admitted to the emergency room. Among them, 20 individuals had a negative result for the SARS-CoV-2 rRT-PCR test, and 39 did not require the immediate initiation of invasive mechanical ventilation. Informed consent could not be obtained from 19 patients. We thus included forty-five patients who provided informed consent for participating in this study.

Of those, the mean age was 57.73 ±18.64 years, and 66.67% were men. A total of 42% had hypertension and 24% had diabetes. Median baseline serum creatinine (SCr) was 0.65 mg/dL (interquartile range (IQR), 0.43–0.79 mg/dL) and SCr at admission was 1.04 mg/dL (±0.49 mg/dL). Other baseline laboratory values and kidney damage biomarkers are shown in Table 1. The SOFA score (Sequential Organ Failure Assessment) on admission was 8 points (8–9 points), vasoactive drugs were necessary in 42% of patients, and 71.1% required IMV in prone position. The initial analysis of arterial blood gases showed a mean PaO2/FiO2 index of 132 (± 58.07), mean pH of 7.29 (± 0.11), mean pCO_2_ of 52.34 mmHg, and mean bicarbonate of 22.2 (IQR 20.3–23.35) mmol/L.

Development of AKI during hospital stay was almost universal (93.3%), but only twenty-three (51.1%) presented pAKI in the first 48 h. In-hospital mortality was 48.9%. Twenty-three patients (51.1%) were extubated during hospital stay, with a median IMV duration of 17 days (IQR 9–48 days).

### 3.2. Characteristics of Patients with MAKEs at 30 Days

**Figure 1:**
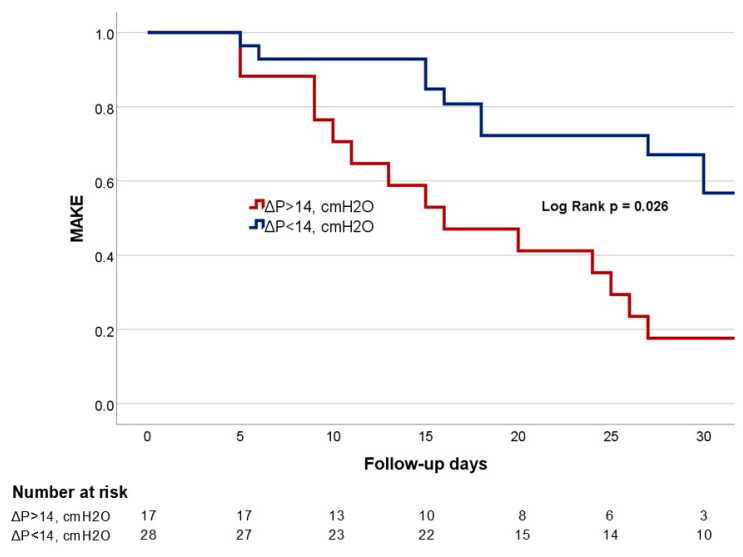
Kaplan–Meier survival curves. Time to death for driving pressure (ΔP) > 14 cmH_2_O group (red line) vs. ΔP < 14 cmH_2_O (blue line). Time 0 corresponded to hospital admission. All patients were censored for 30 days. Patients who were discharged alive before 30 days were treated as still as risk and not censored at discharge.

After compliance-guided PEEP titration (time 0), ventilator settings were higher in the MAKE group, including peak inspiratory pressure (Pmax): 29.07 (±5.56) cmH_2_O in the MAKE group vs. 24 (±5.71) cmH_2_O in the no-MAKE group, *p* = 0.008; Pplat: 25.94 (±4.73) cmH_2_O in the MAKE group vs. 21.64 (±4.09) cmH_2_O in the no-MAKE group, *p* = 0.005; and ΔP: 15 (IQR = 12–20) cmH_2_O in the MAKE group vs. 12 (IQR = 10–13) cmH_2_O in the no-MAKE group, *p* = 0.006. On the other hand, the MAKE group had lower static lung compliance (Cstat): 25.32 mL/cmH_2_O (±8.19) vs. 37.67 mL/cmH_2_O (±8.82) in the no-MAKE group, *p* < 0.001. Likewise, MAKEs were higher in the group of patients with ΔP > 14 cmH_2_O: 16 (51.6%) in the MAKE group vs. 1 (7.14%) in the no-MAKE group, *p* = 0.007. Survival curves of both groups decreased with a similar rate on the first days in hospital, but since day 10, this decrease was steeper in the group of patients with ΔP > 14 cmH_2_O (*p* = 0.026) (Figure 1).

To further characterize ventilatory behavior beyond the baseline, parameters were evaluated at 48 h and 120 h (time 2) after the initiation of mechanical ventilation, comparing patients who developed MAKEs with those who did not.

At 48 h (n = 35), patients in the MAKE group exhibited significantly higher Pmax compared to those without MAKEs (27.5 ± 3.91 vs. 24.38 ± 4.15 cmH_2_O; *p* = 0.033), as well as higher Pplat (24.36 ± 3.67 vs. 21.46 ± 4.05 cmH_2_O; *p* = 0.037). ΔP was also significantly greater in the MAKE group (14 [12,13,14,15,16,17] vs. 11 [9,11,12,13] cmH_2_O; *p* = 0.009), whereas Cstat was notably lower (26.53 ± 8.25 vs. 37.12 ± 9.36 mL/cmH_2_O; *p* = 0.001). No significant differences were observed in tidal volume or PEEP.

At 120 h (n = 32), a similar pattern persisted. The MAKE group continued to demonstrate a significantly higher ΔP (15 [13,14,15,16,17,18,19] vs. 12 [9,11,12,13,14,15] cmH_2_O; *p* = 0.046) and lower Cstat (26.05 ± 9.25 vs. 36.67 ± 10.41 mL/cmH_2_O; *p* = 0.006). Although tidal volume (394.33 ± 77.94 vs. 443.55 ± 58.48 mL; *p* = 0.076) and Pplat (24.62 ± 4.61 vs. 22 ± 5.37 cmH_2_O; *p* = 0.159) were lower in the no-MAKE group, these differences did not reach statistical significance.

Traditional kidney biomarkers were higher in the MAKE group, including SCr 0.95 (IQR = 0.75–1.36) mg/dL in the MAKE group vs. 0.73 (IQR = 0.62–1.01) mg/dL in the no-MAKE group, *p* = 0.041 and BUN: 28 (IQR = 20–35) mg/dL in the MAKE group vs. 20 (IQR = 16–26) mg/dL in the no-MAKE group, *p* = 0.020. Urinary kidney injury biomarkers N-Gal and IL-6 had higher elevations in the MAKE group vs. the no-MAKE group: 50.2 (IQR = 9.9–110.7) ng/dL vs. 10.95 (IQR = 7.4–24-7) ng/dL, *p* = 0.042 and 1.59 pg/mL (IQR 0.39–2.38) vs. 0.36 pg/mL (IQR 0.18–0.86) *p* = 0.024, respectively. The urinary product of the TIMP-2 x IGFBP-7 level did not differ among the MAKE vs. no-MAKE groups.

Patients who developed MAKEs presented with lower oxygen saturation (91% vs. 92.5% *p* = 0.042) and lower pH value (7.27 vs. 7.36 *p* = 0.009) levels compared to the no-MAKE group on admission.

### 3.3. Characteristics of Patients with Persistent Acute Kidney Injury (pAKI)

Serum creatinine at admission was higher in patients with pAKI, in contrast with patients without pAKI, 1.23 (IQR= 0.93–1.53) mg/dL vs. 0.76 (IQR = 0.62–0.95) mg/dL, *p* = 0.002, respectively. Urinary kidney biomarkers were higher in the group with pAKI, including N-Gal 57.1 (IQR = 19–121.3) ng/mL in the group with pAKI vs. 11.1 (IQR = 6.4–26.6) ng/mL in the group without pAKI, *p* = 0.007 and IL-6: 1.73 (IQR = 0.37–4.33) pg/mL in the group with pAKI vs. 0.48 (IQR = 0.29–1.59) pg/mL in the group without pAKI, *p* = 0.076. Other biomarkers like IGFBP-7, TIMP-2, and IGFBP-7 x TIMP-2 did not differ among groups.

### 3.4. Risk Factors for MAKEs: Multivariable and Sensitivity Analysis

The univariate analysis indicated that patients with MAKEs at 30 days were older (unadjusted odds ratio (ORs) = 1.07, 95% CI =1.02–1.12, *p* = 0.003), had a history of type 2 diabetes (ORs = 6.19, 95% CI 0.70–54.15, *p* = 0.099), had higher urinary IL-6 (ORs = 2.13, 95% CI = 0.95–4.75, *p* = 0.064), had higher urinary N-Gal (ORs = 1.01, 95% CI = 0.99–1.04, *p* = 0.089), and had higher ΔP (ORs = 1.36, 95% CI = 1.06–1.74, *p* = 0.013). After adjusting for possible confounding variables, the multivariate analysis indicated that age (adjusted odds ratio (aORs) = 1.23, 95% CI =1.00–1.22, *p* = 0.038) and higher ΔP (aORs = 1.62, 95% CI = 1.01–2.60, *p* = 0.043) were risk factors (Table 2).

In the sensitivity analysis using bootstrapping with 5000 resamples, both driving pressure (ΔP) and age remained independently associated with the development of MAKEs. In particular, ΔP showed a significant association (B = 0.332, 95% bias-corrected and accelerated [BCa] CI: 0.072–61.499, *p* = 0.002), as did age (B = 0.067, 95% BCa CI: −0.007–1.372, *p* = 0.010).

### 3.5. Risk Factor for Persistent Acute Kidney Injury (pAKI)

The univariate analysis indicated that patients with pAKI were older (ORs = 1.06, 95% CI = 1.02–1.10, *p* = 0.005) with higher procalcitonin (ORs = 4.08, 95% CI = 0.70–23.7, *p* = 0.117) and with N-Gal > 40 ng/mL (ORs = 8.43, 95% CI = 2.12–33.61, *p* = 0.002). After adjusting for possible confounding variables, the multivariate analysis indicated that age (aORs = 1.05, 95% CI 1.00–1.10, *p* = 0.038) and N-Gal >40 ng/mL (aORs = 8.54, 95% CI 1.75–41.65, *p* = 0.008) were risk factors for pAKI (Table 3).

## 4. Discussion

Acute kidney injury (AKI) is a frequent and severe complication in patients with ARDS, particularly in the setting of SARS-CoV-2 pneumonia [1]. Early reports indicated that up to 52% of patients with COVID-19-related ARDS requiring mechanical ventilation developed AKI within the first 24 h of intubation [5]. The severity and persistence of AKI have been consistently associated with increased mortality in this population [21], underscoring the importance of identifying modifiable risk factors to prevent kidney damage.

Several studies in non-COVID-19 ARDS populations have established a robust association between elevated driving pressure (ΔP) and poor clinical outcomes, including mortality and lung injury [20,22,23]. ΔP, calculated as the difference between Pplat and PEEP, captures the interaction between tidal volume and Cstat and is therefore a dynamic marker of lung stress and strain [24]. Unlike PEEP or tidal volume alone, ΔP provides a more integrated measure of mechanical burden and has been shown to correlate more strongly with adverse outcomes [20,22]. Amato et al. analyzed trials of MV involving patients with ARDS and found a strong association between ΔP and mortality [relative risk of death (RR), 1.36; 95% CI, 1.17 to 1.58; *p* < 0.001]. Furthermore, they described that for every one standard increment in ΔP, the RR of mortality increased [1.41 (95% CI:1.31 to 1.51, *p* < 0.001] [21]. In a similar way, the LUNG-SAFE study, a large multinational observational cohort, demonstrated that mortality increased with higher quintiles of ΔP on the first day of ARDS [20]. While this and other large cohort studies have provided valuable insights into the role of ΔP and its effects on outcomes such as mortality, they did not specifically investigate its impact on AKI [20,22].

In our cohort of patients with ARDS secondary to SARS-CoV-2, we observed that elevated ΔP, measured after compliance-guided PEEP titration at the initiation of mechanical ventilation, was independently associated with the occurrence of major adverse kidney events (MAKEs) at 30 days. Likewise, uN-Gal values greater than 40 ng/mL were associated with the development of pAKI.

In our study, despite the implementation of a lung-protective ventilation strategy by the respiratory intensive care team, patients who developed MAKEs exhibited persistently lower Cstat, higher Pplat, and elevated ΔP, indicating stiffer lungs and greater mechanical stress. This mechanical burden likely contributed to ventilator-induced lung injury (VILI), a known trigger of systemic inflammation. In support of this hypothesis, we observed elevated urinary IL-6 levels in the MAKE group, suggesting renal exposure to inflammatory mediators. While IL-6 may also reflect the systemic inflammatory response characteristic of severe COVID-19 [25], its presence in urine points toward an active lung–kidney crosstalk. It is plausible that both VILI [26] and viral inflammation [27] acted synergistically, amplifying cytokine release and promoting distal organ dysfunction [28]. This interplay may help explain the increased risk of adverse kidney outcomes in these patients.

The relationship between high ventilatory pressures and adverse kidney outcomes in patients with ARDS has been well documented over the years. The ARMA study (Acute Respiratory Management of ARDS) was pivotal in demonstrating the benefits of low-tidal-volume ventilation in ARDS, including a reduction in the incidence and duration of AKI [29]. Previous evidence in non-COVID-19 populations has also suggested a potential link between impaired lung mechanics and kidney injury. In a retrospective cohort derived from a large public database, both higher PEEP and lower static compliance (Cstat) were independently associated with the development of AKI [7]. Among these variables, Cstat emerged as a particularly relevant marker, and our findings reinforce this association: patients who developed major adverse kidney events (MAKEs) at 30 days consistently showed lower Cstat values. Importantly, this reduction in compliance was not confined to the initiation of mechanical ventilation but persisted throughout the first 120 h, according to our serial measurements. This sustained low compliance reflects a distinct pulmonary phenotype characterized by stiffness and poor recruitability, with elevated driving pressures from the outset and minimal improvement over time [30]. These patients likely did not benefit from higher PEEP, and may in fact have been harmed by it, due to the associated increase in lung stress.

Although numerous studies have shown the deleterious effects of high PEEP on the risk of AKI in mechanically ventilated patients [8,9,31,32,33], we did not observe a significant association between PEEP levels and the development of MAKEs in our cohort. This finding contrasts with recent retrospective studies in patients with SARS-CoV-2, where higher PEEP was associated with a fivefold increase in the risk of AKI and mortality [32], or where central venous pressure (CVP)—which correlated linearly with PEEP—was linked to persistent AKI within five days of intubation [33].

These discrepancies may be explained by the underlying lung mechanics of our cohort. Patients who developed MAKEs exhibited persistently low Cstat, suggesting a rigid, non-recruitable lung phenotype. In such cases, increasing PEEP is unlikely to improve alveolar recruitment and may instead lead to overdistension, increased pulmonary vascular resistance, and hemodynamic compromise [25,34]. This dual mechanism—anterograde effects reducing cardiac output and retrograde effects causing venous congestion—has been well described as a contributor to kidney injury [8,9,33]. Recent studies further emphasize that the hemodynamic consequences of PEEP are highly dependent on lung recruitability: while compliant lungs can accommodate higher PEEP with minimal circulatory impact, stiff lungs experience a disproportionate increase in vascular resistance and right ventricular strain [34,35].

In this context, the absence of an association between PEEP and MAKEs in our study likely reflects the predominance of a low-recruitability phenotype, where high PEEP may have offered no physiological benefit. Instead, Cstat and driving pressure ΔP—which inherently account for the interplay between tidal volume and compliance—emerged as more informative markers of mechanical stress. Patients who developed MAKEs had higher ΔP at baseline, 48 h, and 120 h, suggesting a sustained mechanical burden despite protective ventilation strategies. These findings highlight the complex and evolving relationship between ventilatory mechanics, hemodynamics, and kidney function and support the use of ΔP and Cstat as dynamic markers that reflect the cumulative impact of lung stress on distant organs over time.

In our secondary endpoint, we hypothesized that uNGal and uTIMP-2/IGFBP7 might be correlated with the duration of AKI. However, we found that only uNGal recorded immediately after compliance-guided PEEP titration was significantly associated with pAKI. Our observations align with previous studies, as urinary N-Gal has consistently demonstrated predictive power for persistent AKI in various clinical contexts, whereas the results with TIMP-2/IGFBP7 remain ambiguous [36,37]. A recent research study evaluated the role of uNGal in an adult population for predicting pAKI and secondarily evaluated MAKEs at 30 and 365 days in a heterogenous adult population. While the prediction models for MAKEs were modest, the AUC for pAKI was 0.74 (95% CI 0.70–0.80), suggesting that the uNGal is a valuable tool for predicting this outcome [36]. A few years ago, a two-center cohort study found that NGal was linked to an increased risk of renal adverse events, with a hazard ratio of 1.34 (95% CI, 1.14–1.57), including progression to AKI stages 2–3 [15].

uNGal is a 25 kDA protein that belongs to the lipocalin family. It is produced in renal epithelial cells and leukocytes in response to tubular injury and systemic inflammation [38]. Its presence and levels in the urine can serve as a biomarker for prediction and differentiation of AKI types [13], prediction of renal non-recovery [39], and prediction of in-hospital mortality and long-term prognostic implications [36].

AKI and ARDS are two of the most severe syndromes in critically ill patients, often leading to complex and detrimental lung–kidney interactions. These syndromes not only share common risk factors but also exacerbate each other’s severity, making their co-occurrence particularly dangerous [25,40]. Given the critical interplay between the lungs and kidneys, we believe that a multifaceted approach is essential for managing these conditions. This approach should include early interventions, starting at key moments like the initiation of mechanical ventilation, to implement strategies aimed at preventing adverse outcomes such as MAKEs [12]. By addressing these issues early, we may improve patient prognosis and reduce the incidence of long-term complications.

### Limitations, Strengths, and Weakness

Our study faced several significant limitations, with the most critical being the small sample size. Furthermore, patient recruitment was confined to one of the peak months of emergency bed saturation, during which only the most critically ill patients were admitted. As our center served as a referral hospital for severe cases, this inevitably led to a selection bias, possibly skewing our findings toward the most extreme cases of ARDS and AKI. Moreover, while standardized definitions of AKI rely on serum creatinine (sCr) and urine output, we encountered challenges in accessing complete urinary volume records due to restrictions in nursing documentation within COVID-19 areas. This limitation is particularly significant as it meant that AKI diagnosis could not incorporate urinary output, and sCr was not adjusted for fluid balance, both of which could impact the accuracy of the AKI classification. Additionally, renal function status prior to hospitalization was only determined through patient interviews, where we directly inquired about the presence of chronic kidney disease (CKD), further adding to the potential for misclassification or incomplete data. On the other hand, the main strength of our study lies in the homogeneity of our cohort. All patients were admitted with severe ARDS and required mechanical ventilation within the first 24 h of their emergency room admission. This allowed us to standardize the timing and collection of key ventilator parameters and urine samples, which were gathered at a critical moment immediately following the initiation of mechanical ventilation after PEEP-guided titration. This protocolized approach ensured consistency in terms of data collection and enhanced the reliability of our findings, particularly in linking ventilatory parameters with the development of MAKEs. Future studies could benefit from incorporating repeated measures on consecutive days to track the trajectory of ventilator parameters and urinary biomarkers. This approach could enhance the phenotyping of these patients, providing a more detailed understanding of how these factors evolve over time and their relationship with outcomes like MAKEs.

## 5. Conclusions

Our findings highlight the critical role played by driving pressure (ΔP) and early deviations from protective ventilation in the development of adverse kidney events. Patients who developed MAKEs showed a rigid lung phenotype, with persistently low static compliance (Cstat) and elevated ΔP during the first five days of mechanical ventilation. This pattern suggests poor lung recruitability and a higher risk of ventilator-induced lung injury (VILI). The elevated urinary levels of IL-6 and N-Gal in these patients support a link between mechanical stress, inflammation, and kidney damage. These results emphasize the need for ventilatory strategies tailored to individual lung mechanics and for the ongoing monitoring of both ventilator parameters and biomarkers to reduce kidney injury in this high-risk population.

## Figures and Tables

**Table 1:** General characteristics.

Characteristics	Overall n = 45	MAKE–30 Days n = 31	No-MAKE–30 Days n = 14	*p*-Value
Age, years ^γ^	57.73 (18.64)	63.94 (15.98)	44 (17.09)	**<0.01**
Men [n] ^ø^	30 (66.67)	18 (58.06)	12 (85.71)	0.094
Weight, kg ^γ^	77.22 (16.25)	76.42 (14.76)	79 (19.66)	0.627
Height, cm ^γ^	163.7 (11.39)	161.55 (10.33)	168.57 (12.51)	0.055
BMI, kg/m^2 γ^	28.69 (5.42)	29.2 (5.77)	27.58 (4.56)	0.358
Comorbidities				
Hypertension [n] ^ø^	19 (42.22)	13 (41.94)	6 (42.86)	1.000
Diabetes [n] ^ø^	10 (24.44)	10 (32.26)	1 (7.14)	0.132
Heart disease ^ø^	3 (6.67)	3 (9.68)	0 (0)	0.541
HIV ^ø^	3 (6.67)	1 (3.23)	2 (14.29)	0.224
Other comorbidities ^ø^	2 (4.44)	1 (3.23)	1 (7.14)	0.530
Laboratories				
Leucocytes, 10^3^/mm^3 γ^	13.9 (9.65–16.6)	15.7 (10.3–17.4)	11.49 (8.86–14.15)	0.148
Neutrophils, 10^3^/mm^3 γ^	12.5 (8–15.5)	13.1 (8–16)	10.65 (8–13)	0.239
Lymphocytes, 10^3^/mm^3 γ^	0.7 (0.5–1)	0.7 (0.4–1)	0.8 (0.5–1.2)	0.438
Hemoglobin, g/dL ^γ^	14.37 (1.68)	14.33 (1.77)	14.5 (1.51)	0.754
Hematocrit, % ^γ^	43.07 (5.31)	42.93 (5.8)	43.4 (4.22)	0.785
Platelets, 10^3^/mm^3 γ^	273.1 (98.35)	283.51 (105.84)	250.26 (77.92)	0.299
Sodium, mmol/L ^γ^	137.2 (5.71)	137.03 (6.3)	137.57 (4.33)	0.773
Potassium, mmol/L ^γ^	4.31 (0.66)	4.35 (0.75)	4.24 (0.41)	0.614
Chloride, mmol/L ^γ^	102 (99–105)	102 (97–106)	102.5 (100–105)	0.768
Calcium, mg/dL ^γ^	8.06 (0.57)	8.04 (0.61)	8.11 (0.51)	0.715
Magnesium, mg/dL ^γ^	2.2 (1.9–2.4)	2.2 (1.9–2.6)	2.2 (1.9–2.3)	0.459
Phosphate, mg/dL ^γ^	3.8 (3.1–4.5)	4 (2.9–5.2)	3.7 (3.2–3.9)	0.244
Glucose, mg/dL ^γ^	138 (112–188)	149 (127–206)	108 (85–138)	**0.006**
HbA1C, % ^γ^	5.855 (5.5–7.3)	6.16 (5.77–7.62)	5.57 (5.17–5.59)	0.066
BUN mg/dL ^γ^	23 (18–31)	28 (20–35)	20 (16–26)	**0.020**
Creatinine at admission mg/dL ^γ^	0.95 (0.695–1.285)	0.95 (0.75–1.36)	0.73 (0.62–1.01)	**0.041**
Baseline creatinine, mg/dL ^ρ^	0.65 (0.43–0.79)	0.69 (0.44–0.87)	0.53 (0.36–0.7)	**0.050**
LDH, U/L ^γ^	635.5 (279.6)	661.3 (267.82)	578.37 (306.85)	0.363
CPK, U/L ^γ^	85 (38–191)	99 (38–191)	71.5 (38–205)	0.893
ESR, mm/hr ^ρ^	33 (20–50)	34 (18–50)	31.5 (27–44)	0.773
Ferritin, ng/mL ^ρ^	1135.11 (453–1914.19)	1158.6 (433.52–1855)	1122.56 (701.67–2923)	0.694
D-Dimer, µg/mL ^ρ^	2.31 (0.475–6.065)	2.62 (0.59–6)	1.08 (0.44–6.13)	0.495
Procalcitonin ng/mL ^γ^	0.21 (0.12–0.4)	0.21 (0.11–0.73)	0.24 (0.14–0.38)	0.893
Procalcitonin > 0.5 ng/mL ^ø^	10 (22.22)	8 (25.8)	2 (14.29)	0.469
C-Reactive protein, mg/dL ^γ^	17.05 (7.94)	17.49 (7.31)	16.04 (9.5)	0.587
Troponin-I, pg/mL ^ρ^	16.3 (4.8–64.6)	44.5 (9.3–72.5)	2.9 (2.2–6.85)	**<0.01**
BNP, pg/mL^ρ^	62.9 (30.2–197.2)	102 (40.8–252.8)	36.5 (16.5–63.1)	**0.009**
Fibrinogen, mg/dL ^γ^	691.5 (608–768.5)	693 (605–768)	690 (613–785)	0.976
Critical Care Variables				
pH ^γ^	7.29 (0.11)	7.27 (0.12)	7.36 (0.08)	**0.009**
pO_2_, mmHg ^γ^	66.8 (58.2–80)	64.5 (57–85)	70 (63–78.5)	0.229
pCO_2_, mmHg ^γ^	49 (39–58)	50 (39–68)	44.4 (37–55)	0.117
PaO_2_/FiO_2_, mmHg ^γ^	132.0 (58.07)	122.12 (62.82)	154.14 (39.37)	**0.045**
SpO_2_	92 (88.5–94.8)	91 (84–94.8)	92.5 (92–97)	**0.042**
HCO_3_-, mmol/L ^ρ^	22.2 (20.3–24.8)	21.9 (19.8–23.3)	23 (22–25)	0.364
Fluid balance, mL ^γ^	1051 (512–1685)	1230 (540–1826)	720.5 (380.2–1237.6)	0.159
SOFA^ρ^	8 (8–9)	8 (8–9)	8 (8–9)	0.967
MAP, mmHg ^γ^	74 (71–78)	75 (71.5–78)	72 (69–80)	0.524
HR, bpm ^ρ^	80 (70–100)	81 (72–101)	77 (67–87)	0.569
Vasoactive drugs ^ø^	18 (40)	13 (41.94)	5 (35.71)	0.753
Prone-position ventilation ^ø^	32 (71.11)	25 (80.65)	7 (50)	0.072
Urinary Kidney Biomarkers				
IGFBP7, ng/mL ^γ^	11.79 (6.63–30.77)	14.32 (6.71–45.03)	7.79 (5.69–12.86)	**0.047**
TIMP-2, ng/mL ^γ^	4.79 (1.79–10.53)	4.43 (1.95–10.35)	5.39 (1.05–13.48)	0.980
IL-6, pg/mL ^ρ^	0.72 (0.31–1.91)	1.59 (0.39–2.38)	0.36 (0.18–0.86)	**0.024**
[(TIMP-2)(IGFBP-7)]/1000 ^ρ^	0.05 (0.01–0.3)	0.1 (0.02–0.33)	0.04 (0.01–0.16)	0.226
N-Gal ^ρ^	24.7 (8.8–82.2)	50.2 (9.9–110.7)	10.95 (7.4–24.7)	**0.042**
Ventilatory parameters after compliance-guided PEEP titration				
PEEP, cmH_2_O ^ρ^	8 (8–12)	8 (7–12)	10 (8–12)	0.447
Tidal Volume, mL ^γ^	394.5 (64.97)	383.93 (63.46)	418.23 (64.39)	0.115
Pmax, cmH_2_O ^γ^	27.41 (6.04)	29.07 (5.56)	24 (5.71)	**0.008**
Pplat, cmH_2_O ^γ^	24.6 (4.91)	25.94 (4.73)	21.64 (4.09)	**0.005**
ΔP, cmH_2_O ^γ^	13 (11–18)	15 (12–20)	12 (10–13)	**0.006**
ΔP > 14, cmH_2_O ^ø^	17 (37.78)	16 (51.61)	1 (7.14)	**0.007**
Cstat, ml/cmH_2_O ^γ^	29.02 (9.68)	25.37 (7.9)	37.14 (8.42)	**< 0.001**
Outcomes				
Extubation ^ø^	23 (51.11)	10 (32.26)	13 (92.86)	**<0.01**
Days on IMV ^ø^	17 (9–48)	22 (10–54)	16 (9–42)	0.343
Death ^ø^	22 (48.89)	21 (67.74)	1 (7.14)	**<0.01**

Abbreviations: BMI, body mass index; HIV, human immunodeficiency virus; HbA1C, glycosylated hemoglobin; BUN, blood urea nitrogen; LDH, lactic dehydrogenase; CPK, creatine phosphokinase; ESR, erythrocyte sedimentation rate; BNP, B-type natriuretic peptide; pO_2_, partial pressure of oxygen; pCO_2_, partial pressure of carbon dioxide; HCO_3_−, sodium bicarbonate; IGFBP7, insulin-like growth factor binding protein 7; TIMP-2, tissue inhibitor of metalloproteinases-2; IL-6, interleukin-6; ((TIMP2)(IGFBP7)]/1000, (tissue inhibitor of metalloproteinases-2)(insulin-like growth factor binding protein 7))/100; NGAL, neutrophil gelatinase-associated lipocalin; SpO2, oxygen saturation; MAP, mean arterial pressure; HR, heart rate; PEEP, positive end-expiratory pressure; Pmax, maximum airway pressure; Pplat, plateau pressure; ΔP, driving pressure; Cstat, static lung compliance; PaO2/FiO2, partial pressure of oxygen/inspired fraction of oxygen; SOFA, Sequential Organ Failure Assessment; IMV, invasive mechanical ventilation. Comparisons of MAKE vs. no-MAKE group were made using Student’s T-test (γ), where their data are expressed as mean (standard deviation); Mann–Whitney U-test for continuous variables (ρ), where their data are expressed as median (interquartile range); and Fisher test’s for categorical variables (ø). Bold values denote statistical significance at the *p* ≤ 0.05 level.

**Table 2:** Risk factors for MAKEs at 30 days.

Variables	Unadjusted ORs (95% CI)	*p*-Value	Adjusted ORs (95% CI)	*p*-Value
Age, years	1.07 (1.02–1.12)	**0.003**	1.23 (1.00–1.22)	**0.038**
Male	0.23 (0.04–1.21)	0.083	0.15 (0.03–5.78)	0.432
Diabetes	6.19 (0.70–54.15)	0.099	0.62 (0.02–16.77)	0.619
ΔP, cmH_2_O	1.36 (1.06–1.74)	**0.013**	1.62 (1.01–2.60)	**0.043**
Urinary N-Gal	1.01 (0.99–1.04	**0.089**	1.01 (0.98–1.05)	0.323
Urinary IL-6, pg/ml	2.13 (0.95–4.75)	**0.064**	1.81 (0.62–5.33)	0.281

Abbreviations: ORs, odds ratio; IL-6, interleukin-6; N-Gal, neutrophil gelatinase-associated lipocalin; ΔP, driving pressure. Model is adjusted to serum leucocytes >12,000/μL, SOFA score, and serum C-reactive protein. Bold values denote statistical significance at the *p* ≤ 0.10 level in univariate analysis and *p* ≤ 0.05 in multivariate analysis.

**Table 3:** Risk factors for persistent AKI.

Variables	Unadjusted ORs (95% CI)	*p*-Value	Adjusted ORs (95% CI)	*p*-Value
Age, years	1.06 (1.02–1.10)	**0.005**	1.05 (1.00–1.10)	**0.038**
Men	0.58 (1.66–2.55)	0.401	0.66 (0.12–3.58)	0.639
Procalcitonin, ng/ml	4.08 (0.70–23.7)	0.117	2.61 (0.40–16.87)	0.311
Urinary N-Gal > 40 ng/mL	8.43 (2.11–33.61)	**0.002**	8.54 (1.75–41.65)	**0.008**

Abbreviations: ORs, odds ratio; IMV, N-Gal, neutrophil gelatinase-associated with lipocalin. Bold values denote statistical significance at the *p* ≤ 0.20 level in univariate analysis and *p* ≤ 0.05 in multivariate analysis.

## Data Availability

All data generated and analyzed during this study were included in a Supplementary Materials File (S1 File Raw Data).

## Supplementary Materials

The following supporting information can be downloaded at: https://www.mdpi.com/article/10.3390/jcm14082783/s1, S1 File Raw Data.
